# Early-life gut microbiota associates with allergic rhinitis during 13-year follow-up in a Finnish probiotic intervention cohort

**DOI:** 10.1128/spectrum.04135-23

**Published:** 2024-04-30

**Authors:** Sampo Kallio, Ching Jian, Katri Korpela, Anna Kaarina Kukkonen, Anne Salonen, Erkki Savilahti, Mikael Kuitunen, Willem M. de Vos

**Affiliations:** 1 Children’s Hospital, University of Helsinki and Helsinki University Hospital, Helsinki, Finland; 2 Human Microbiome Research Program, Faculty of Medicine, University of Helsinki, Helsinki, Finland; 3 Laboratory of Microbiology, Wageningen University, Wageningen, the Netherlands; Huazhong University of Science and Technology, Wuhan, Hubei, China

**Keywords:** gut microbiota, probiotics, asthma/allergy, rhinitis

## Abstract

Perinatal and early-life factors reported to affect risk of allergic diseases may be mediated by changes in the gut microbiota. Here, we explored the associations between the infant gut microbiota and allergic morbidity in childhood until 13 years of age in a subgroup of the FLORA probiotic intervention cohort. A mixture of four probiotic strains with galacto-oligosaccharides was administrated to the mothers from the 36th week of the pregnancy and later to their infants until 6 months of age. The infants were monitored for the manifestations of atopic eczema, food allergy, allergic rhinitis, and asthma by a pediatrician at 2 and 5 years of age; the allergic status was subsequently verified by a questionnaire at 10 and 13 years of age. The fecal microbiota at 3 months was profiled by 16S rRNA amplicon sequencing targeting the V3-V4 region, with and without adjusting for potentially important early-life factors. Overall, the positive diagnosis for allergic rhinitis between 2 and 13 years was associated with microbiota composition both in non-adjusted and adjusted models. This association was more pronounced in children born to one parent with confirmed atopic diseases compared to those who had two atopic parents and was characterized by a lower relative abundance of *Bifidobacterium* and *Escherichia/Shigella spp*. and a higher proportion of *Bacteroides*. While the probiotic and galacto-oligosaccharides intervention in the entire cohort was previously shown to reduce the prevalence of eczema to a certain extent, no associations were found between the 3-month gut microbiota and childhood eczema in the studied sub-cohort.

**IMPORTANCE:**

Allergic diseases have increased in prevalence during the past decades globally. Although probiotics have been considered a promising strategy for preventing certain allergy related symptoms, studies connecting the infant gut microbiota and later life allergic morbidity in various populations remain limited. The present study supports an association between the infant microbiota and allergic morbidity after first years of life, which has been rarely examined.

**CLINICAL TRIALS:**

Registered at ClinicalTrials.gov (NCT00298337).

## INTRODUCTION

Allergic diseases such as asthma, allergic rhinitis (or hay fever), food allergy, and atopic dermatitis (or eczema) share common etiological mechanisms characterized by an exaggerated immune response and the elevated production of allergen-specific immunoglobulin E (IgE) (1) and are similarly associated with the risk factors linked to altered gut microbiota development during infancy (2). The gut microbiota primes the immune system notably in early life via the extensive immune-microbiota crosstalk (3). For instance, it has been hypothesized that reduced proportions of butyrate-producing gut bacteria lead to reduced regulatory T cell populations, increasing the risk of allergic disease (4, 5). Intestinal bifidobacteria that are abundantly present in infants have been reported to modulate T cells in a strain-dependent way (6). Microbial metabolites including short chain fatty acids have been considered pivotal in maintaining intestinal epithelial barrier function and mucosal immunity (7). Therefore, it is not surprising that increasing evidence is pointing to specific infant gut microbiota signatures predictive of allergic morbidity during childhood.

The reported links between the gut microbiota and the development of allergy vary across cohorts and populations. For example, Swedish children diagnosed with atopic disease at 5 years harbored less *Bifidobacterium* and *Lactobacillus* in infancy (8). high relative proportion of *Bifidobacterium* was associated with healthy phenotypes while that of *Klebsiella* was associated with allergic diseases until 3 years of age in the Singaporean GUSTO (Growing Up in Singapore Towards healthy Outcomes) cohort (9). On the other hand, in a small cohort of 30 Taiwanese twins and 14 matched singletons, the relative abundance of *Ruminococcus gnavus* and *Lachnospiraceae* was associated with allergic diseases at 3 years of age (10). An altered microbiota community structure, such as reduced microbiota diversity in infancy, was associated with allergic rhinitis and sensitization, but not asthma or atopic sensitization at 6 years in a large Danish Copenhagen Prospective Study on Asthma in Childhood (COPSAC) cohort of over 400 infants (11). Among allergic diseases, asthma has gained special interest due to its major interference with daily activities. In the Canadian CHILD study of 319 infants, the relative levels of *Lachnospira*, *Veillonella*, *Faecalibacterium,* and *Rothia* were found to be decreased in children with asthma (12). A recent comprehensive study of the COPSAC 2010 cohort of 690 infants reported that the relative abundances of *Bifidobacterium*, *Roseburia*, *Alistipes*, *Ruminococcus*, and *Dialister* at 5 years strongly and negatively correlated with asthma, while these associations were more pronounced in the children of asthmatic mothers (13). The connection among the early-life gut microbiota, development of oral tolerance, and food allergy has also been explored (14). While suggesting a gut microbiota component in the pathogenesis of allergy, most of the available studies are based on general or low-risk populations. Observational studies in high-risk children in a population with relatively homogeneous genetics, such as the Finnish population (15) may offer new insights into the role of the gut microbiota in allergic diseases that have a salient genetic component.

Probiotics are thought to positively influence the composition of gut microbiota and interact with different immune cells, promoting a signaling cascade in terms of pro- and anti-inflammatory cytokine release and thus modulating immune functions (16). Probiotics have been mainly prescribed for intestinal disorders in children and adults, such as diarrhea, colitis, pouchitis, necrotizing enterocolitis, and acute gastroenteritis (16), but their potential immunoregulatory function has also been tested in managing allergy (16). In a randomized, double-blind, placebo-controlled trial enrolling 1,018 Finnish children with a high risk of allergy (FLORA trial), we previously reported that the treatment with a mixture of specific strains of *Lactobacillus* and *Bifidobacterium* with galacto-oligosaccharides reduced the prevalence of eczema at 2 years (17). In the caesarean-delivered sub-group, this effect persisted up to 13 years (18). Here, we capitalized on this well-phenotyped cohort to explore the potential associations among early-life factors, the infant gut microbiota at 3 months, and the prevalence of allergic diseases during the 13-year follow-up timeframe. We hypothesized that the children, who developed allergic diseases later in life, had an altered gut microbiota compared to healthy controls in early infancy.

## MATERIALS AND METHODS

### Study design and participants

This investigation was carried out in a sub-cohort (*n* = 383) of the FLORA cohort. The recruitment for the FLORA study was initially carried out among pregnant women carrying a child with a high risk for allergic disease. The population lived in Southern Finland. The high allergy risk was defined so that at least one of the parents has had doctor-diagnosed asthma, allergic rhinitis, or atopic eczema. The participating women (*n* = 1,223) were randomized to receive a mixture of live micro-organisms and oligosaccharides or placebo in a double-blind setting. The treatment product comprised a capsule containing freeze-dried *Lactobacillus rhamnosus* GG [American Type Culture Collection (ATCC) 53103; 5 × 10^9^ colony-forming units, CFU], *L. rhamnosus* LC705 [Deutsche Sammlung von Mikroorganismen (DSM) 7061; 5 × 10^9^ CFU], *Bifidobacterium breve* Bb99 (DSM 13692; 2 × 10^8^ CFU), and *Propionibacterium freudenreichii* subsp. *Shermanii* JS (DSM 7076; 2 × 10^9^ CFU). Probiotic strains were selected based on the state-of-the-art knowledge at the time on the safety and potential functional use of individual strains and their combinations. Starting from 36 weeks of gestation, the mothers in treatment group received one capsule twice a day until delivery, and thereafter the infants received the same capsule opened and mixed with 20 drops of syrup containing 0.8 g of prebiotic galacto-oligosaccharides once daily until 6 months of age. Mothers and infants in the placebo group received products that appeared similar without micro-organisms or oligosaccharides. Exclusion criteria were birth at less than 37 weeks of gestation, major malformations, and the second born of twins. Because of exclusion criteria and drop-out, there were 1,018 intention-to-treat infants. The treatment protocol has been previously explained in more detail (17) and the effect of the probiotic treatment for the allergy-prevention at 2, 5, 10 at 13 years has been previously reported (17–20).

### Cohort follow-up

At 3 months, 872 participants provided fecal sample. Some of those have been used in previous investigations, so consequently 383 of the samples were available for this investigation representing randomly selected samples. Full examination by a pediatrician was carried out at 2 and 5 years. After the 5-year visit, the participants and investigators were unblinded. At 10- and 13-years, the follow-up was carried out via questionnaires, where the participants were inquired regarding doctor-diagnosed allergies, probiotic use, as well as relevant environmental and lifestyle factors.

### Definitions of disease cases

In the present study, four allergic diseases were investigated, including asthma, eczema, allergic rhinitis, and food allergy. At 2 and 5 years, the diagnosis was based on the evaluation by a research pediatrician using well defined criteria; food allergy by open challenge in infants (21), atopic eczema using United Kingdom (UK) Working Party’s criteria (22) (an itchy skin condition in addition to three or more of the following conditions: familial history of atopic disease, dry skin during the last year, history of eczema, or visible eczema involving typical sites). Asthma was diagnosed by two or more doctor-diagnosed wheezing episodes, persistent cough and exercise-induced symptoms (23), and later also using lung function tests. Allergic rhinitis was diagnosed with a history of two or more than two symptoms of nasal discharge, blockage, and sneeze/itch recurrently during allergen contact and antigen-specific IgE sensitization (24). At 10 and 13 years, the diagnosis was based on self-reports of doctor-diagnosed allergic diseases using questionnaires. The diagnosis was considered positive if a given disease was present in any of the ages mentioned previously.

### Sensitization analysis

Sensitization was determined by skin prick tests (SPTs) at 2 and 5 years and by measuring specific IgE antibodies from blood at 2, 5, and 13 years, as previously described (17–19). SPTs were performed on the forearm to determine reactivity for cat, dog, birch, timothy, mugwort, *Dermatophagoides pteronyssinus* (house dust mite), cow’s milk, egg, wheat, and peanut with commercial solutions (ALK-Abelló, Hørsholm, Denmark, or Stallergenes, Antony, France) or fresh food dilutions with 0.9% sodium chloride. Histamine chloride was used as a positive and glycerin as a negative control. A wheal diameter of 3 mm or greater than the negative control was considered positive. Blood samples were drawn for analyzing specific IgE antibodies against milk, egg white, birch, timothy, (mugwort), cat and dog, peanut, and *D. pteronyssinus* (house dust mite) by using the ImmunoCAP system (Phadia, Uppsala, Sweden) according to the manufacturer’s instructions. ImmunoCAP tests use fluorescently labeled detection antibodies to measure levels of specific IgEs. The detection limit was set to 0.01 kU/L, and a concentration of greater than 0.7 kU/L was considered positive.

### DNA extraction and 16S rRNA gene amplicon sequencing

Fecal samples were collected at home by the participating families and frozen immediately at −20°C before transporting to the laboratory in frozen form on the next day. The samples were then stored at −80°C until DNA extraction. Bacterial DNA was extracted from fecal samples using a modified version of repeated bead beating that efficiently extracts bacterial DNA from both Gram-positive and -negative cocci, as described in detail elsewhere (25). The library preparation was performed essentially according to the protocol by Illumina, except that the 16S rRNA gene amplification and barcoding were performed in a single reaction. The PCR reaction comprised 1 ng/µL template, 1X Phusion Master Mix (ThermoFisher, catalog number: F-531L), 0.25 µM V3-V4 locus-specific primers and 0.375 µM TruSeq dual-index primers. The PCR was run under the following settings: 98°C for 30 s, 27 cycles of 98°C for 10 s, 62°C for 30 s, 72°C for 15 s, and finally 10 min at 72°C, whereafter the samples were stored at 4°C. The PCR clean-up was performed with AMPure XP beads (Beckman Coulter, Copenhagen, Denmark) and confirmation of the correct amplicon size (ca. ~640 base pairs) was performed on a Bioanalyzer DNA 1000 chip (Agilent Technology, Santa Clara, CA, USA). The pooled libraries were sequenced with an Illumina MiSeq or HiSeq2500 in Rapid Run mode (26). Negative control samples were included during sample processing and library preparation to identify and remove potential contaminants as described previously (26).

### Data processing and statistical analysis

Demultiplexed reads after adaptor removal were processed using DADA2 (27) to generate amplicon sequence variants (ASVs). Taxonomic classification was performed using a naïve Bayes classifier against the SILVA 132 reference database (27). Samples had a mean sequencing depth of 53,320 ± 24,150 (mean ± SD) reads.

Permutational multivariate analysis of variance [PERMANOVA; *adonis*2 function in the *vegan* package (28) with 999 permutations based on the Bray–Curtis dissimilarity, robust Aitchison distance (29), or generalized UniFrac distance (30) matrices] was used to identify variables associated with the variation in microbiota composition in univariate and multivariate models. *Adonis*2 was run by “margin,” which calculates the marginal *R*^2^ for each variable after adjusting for the other variables in multivariate models. Principal coordinate analysis (PCoA) plots with the Bray–Curtis dissimilarity were employed to visualize the differences in overall microbiota composition between participants with and without a given allergic disease. Microbiota richness and Shannon diversity index were estimated using the *vegan* package.

Differential abundance testing for bacterial genera was performed using R package Microbiome Multivariable Association with Linear Models (*MaAsLin*2), while adjusting for potential confounders (31). Raw counts were normalized by total sum scaling to generate relative abundances and log-transformed prior to model fitting in *MaAsLin*2; the data were filtered to retain features present in >10% of samples, and the *q*-value (adjusted *P*-value by the Benjamini–Hochberg method) threshold of 0.05 was used for significance. The results were validated using *DESeq2*, which employs a generalized linear model of counts based on a negative binomial distribution, scaled by a normalization factor that accounts for differences in sequencing depth between samples (32); the default *q*-value threshold of 0.05 was used for significance. The consensus results obtained by *MaAsLin*2 and *DESeq*2 were considered significantly differential genera and reported herein.

The *PathModel* function with default settings in the R package *mare* was used to construct an association network among early-life factors, the gut microbiota and rhinitis (33). The *PathModel* function selected appropriate linear models for the identification of significant candidate variables, and then combined them all into one model via the functions *step* and *stepAIC* in the packages *stats* and *MASS*.

Noncount variables (e.g., microbiota diversity and richness) were analyzed with a Wilcoxon signed-rank test or student’s *t*-test for nonnormally distributed and normally distributed variables, respectively. Statistical differences in the proportions of categorical variables between study groups were evaluated using chi-squared tests. *P*-values are corrected for multiple testing when applicable as described above, where *P*- and *q*-values < 0.05 are considered significant.

## RESULTS

### General characterization of the analyzed cohort

A sub-cohort of 383 from the 1,018 infants enrolled in the FLORA study provided fecal samples at 3 months of age and was subjected to gut microbiota profiling by 16S rRNA gene amplicon sequencing. The FLORA trial design and participation during the follow-up are summarized in Fig. S1. Key early-life factors in the whole FLORA cohort and the sub-group analyzed herein are presented in Table 1. Cesarean birth was less common in this sub-cohort (13.6% vs 20%) compared to the rest of the cohort. There was also a smaller but significant difference in birth weight, birth height, and usage of antibiotics during the intervention. Of the 383 infants, 197 (51.4%) were girls. Between 2 and 13 years, there were 60 participants diagnosed with asthma (15.7%), 85 (22.2%) with food allergy, 99 (25.8%) with allergic rhinitis, and 175 (45.7%) with eczema; altogether, 247 (64.5%) of them had at least one of the abovementioned conditions.

**TABLE 1:** Comparison of the present sub-cohort (gut microbiota data available) and the rest of the FLORA cohort

	Microbiota data available	Microbiota data unavailable	*P*-value
	*n* = 383	*n* = 589	
Treatment group (%)	53	48.2	0.1638
Female (%)	51.4	49.7	0.6531
Birth weight (g)	3,541	3,610	0.0324^*a*^
Birth height (cm)	50.4	50.7	0.0349^*a*^
Mothers’ age at labor (y/o)	31.14	31.02	0.7178
Maternal atopy (%)	80.9	80.8	1.0000
Paternal atopy (%)	56.7	59.4	0.4308
Biparental atopy (%)	37.6	40.4	0.4184
Cesarean birth (%)	13.6	20	0.0123^*a*^
Parents with higher education (%)	44.9	47.2	0.2201
Antibiotics during intervention (%)	19.8	16.3	0.0158^*a*^
Siblings > 1 (%)	46	40.6	0.0978

^*a*^ *p* < 0.05.

### Overall gut microbiota composition explained by early-life factors

**FIG 1:**
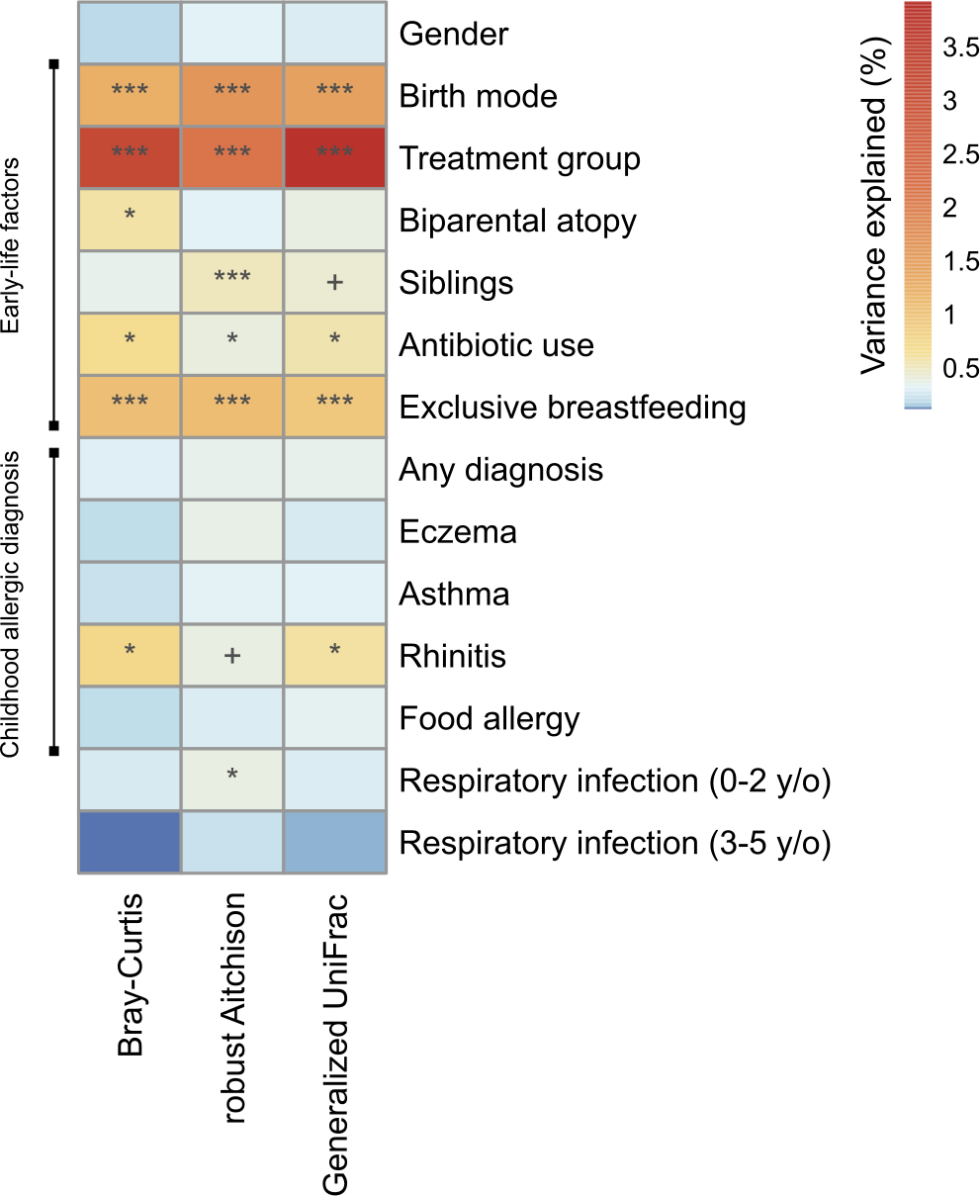
Associations between the overall gut microbiota at 3 months represented by three β-diversity metrics (Bray–Curtis, robust Aitchison and generalized UniFrac), early-life factors, and allergic diagnosis outcomes (2–13 years; see Materials and Methods). Variables significantly associated with microbiota composition measured by more than two β-diversity metrics were considered consistent associations. Variation in the microbiota explained by each variable (%) was derived from the *R*^2^ value in PERMANOVA. + *P* < 0.1; * *P* < 0.05; ** *P* < 0.01; *** *P* < 0.001.

We first examined the associations between overall microbiota structure represented by the Bray–Curtis dissimilarity, robust Aitchison distance, or generalized UniFrac distance matrices and six early-life factors in univariate models (Fig. 1). The early-life factors significantly explained the variance in microbiota composition measured by more than two of the β-diversity metrics were considered consistent associations. Results showed that the gut microbiota at 3 months was mainly associated with birth mode, antibiotic use (0–6 months), and exclusive breastfeeding and probiotic treatment, with probiotic treatment accounting for the largest microbiota variation as expected (Fig. 1).

### Overall gut microbiota composition predictive of childhood allergic diseases

We repeated the univariate association analysis for allergy disease diagnoses as well as respiratory infections during childhood (until 13 years of age). This was to understand whether the early-life microbiota configuration was predictive of allergy- and airway infection-related diseases later in life. Allergic rhinitis was identified as the only disease significantly associated with the 3-month gut microbiota composition (Fig. 1). To confirm the lack of predictability for eczema and asthma, two allergic diseases reported to associate to an altered neonatal gut microbiota as early as 1 month of age (34), we investigated the associations using the available diagnosis of eczema and asthma at 2 years (for eczema) and 5 years (for eczema and asthma) but found no significant associations (Fig. S2). We also performed multivariate analyses for allergic disease diagnoses adjusting for birth mode, probiotic treatment, biparental atopy (whether both parents had atopy), antibiotic use, and exclusive breastfeeding. Allergic rhinitis remained the only outcome that was significantly associated with the gut microbiota variation at 3 months (*P* = 0.015).

**FIG 2:**
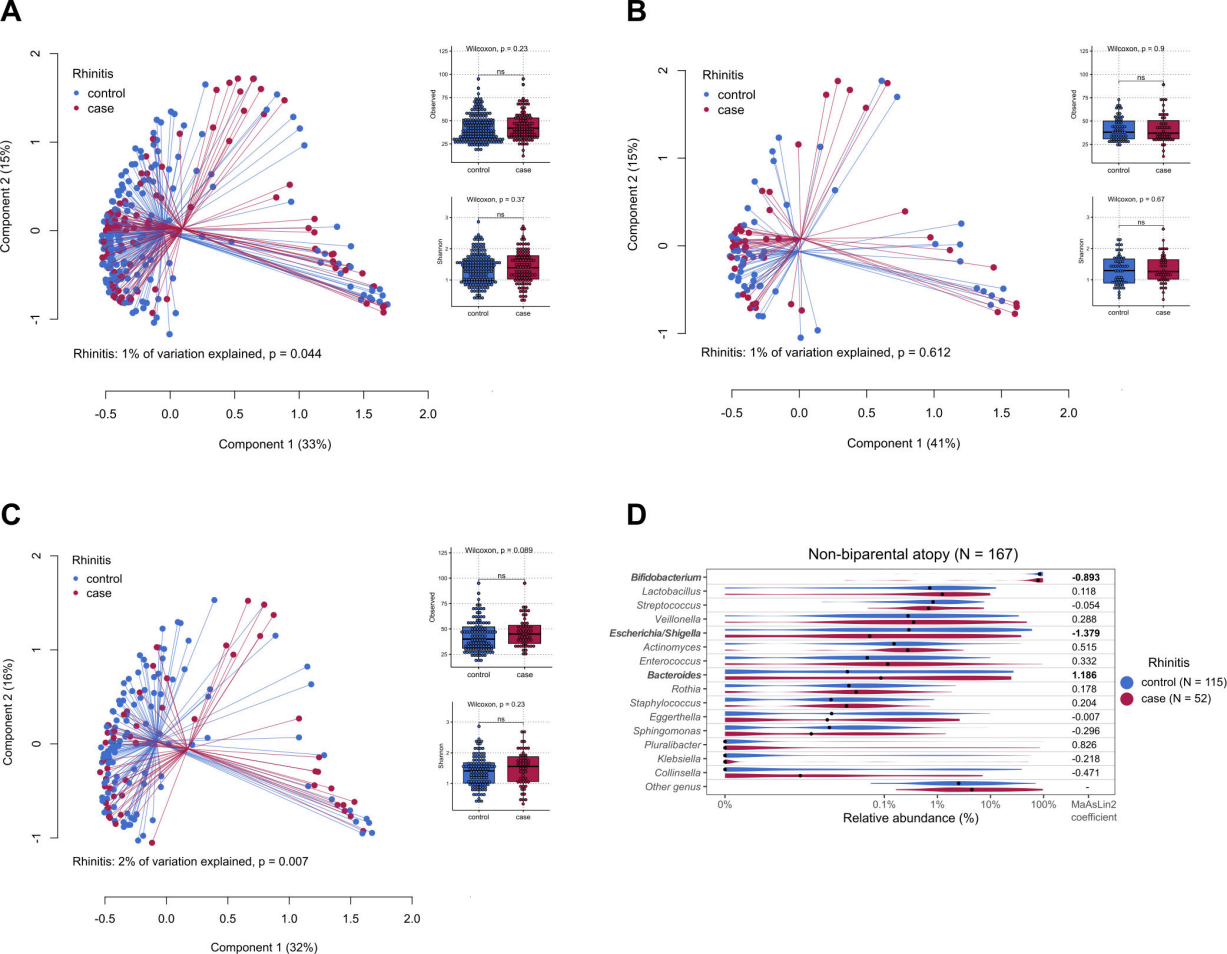
Comparison of microbiota β-diversity visualized by the PCoA plot and *α*-diversity estimated by observed richness and Shannon diversity between children with allergic rhinitis and non-rhinitis controls in the (A) overall group (*N* = 273; case/control = 99/174), (B) biparental atopy (*N* = 106; case/control = 47/59), (C) non-biparental atopy (*N* = 167; case/control = 52/115) sub-group. ns, non-significant. (D) top 15 abundant bacterial genera identified in the non-biparental atopy sub-group. The genera significantly enriched (positive coefficients) or depleted (negative coefficients) in children with rhinitis after controlling for other early-life factors are in bold (see Table S2F for full results).

Sub-group analysis of the disease outcomes was performed stratified by birth mode, probiotic treatment, biparental atopy, antibiotic use, and exclusive breast feeding. A significant difference in the overall gut microbiota composition between participants with and without rhinitis was identified in the non-biparental atopy sub-group (PERMANOVA *P* = 0.007; Fig. 2A through C). Similar findings were found when analyzing a subset of the participants with available aero-IgE testing outcomes (*N* = 174; Fig. S3). Of note, this association was not observed in the infants where both parents had atopy, pointing to a genetic effect. No significant differences in microbiota *α*-diversity (estimated by observed richness and Shannon diversity index) were found in the overall non-biparental atopy or biparental atopy sub-group (Fig. 2A through C). No other significant differences in the overall gut microbiota composition were found to be associated with other allergic diseases or airway infections in other sub-group analyses stratified according to the abovementioned early-life factors.

A comparison of the clinical characteristics between the children with rhinitis and non-rhinitis controls is presented in Table S1. Eczema, asthma, and food allergy were more common in the rhinitis group (*P* < 0.05), suggesting that the positive rhinitis diagnosis likely captured those, who had multiple allergic diseases. Of note, children with rhinitis were more likely to be born to the parents, who both had atopic diseases, that is, biparental atopy (*P* < 0.05; Table S1).

### Taxonomic characteristics of the early-life gut microbiota in childhood allergic diseases

No significant differences in the relative abundance of bacterial genera were found between the participants with and without any allergy diagnosis, asthma, eczema, and food allergy during childhood (*q* > 0.05; Table S2A through D). Focusing on the available diagnosis of eczema at 2 years (Table S3A through B) and eczema and asthma at 5 years (Table S3C through F), we found that the relative abundance of *Haemophilus*, an asthma-associated bacterial genus identified from the airway microbiota (35), was significantly and positively associated with eczema at 5 years (*q* < 0.05; Table S3C through D).

We next focused on the children with and without rhinitis (*N* = 273) in relation to parent’s atopy status, as the β-diversity analysis hinted taxonomic differences in the gut microbiota (Fig. 2A through C). Rhinitis was significantly and negatively associated with the relative abundance of *Escherichia*/*Shigella* (*q* < 0.05; Table S2E). This association was more pronounced in the non-biparental atopy group (*N* = 167), where a significantly reduced relative abundance of *Bifidobacterium* and a significantly increased relative proportion of *Bacteroides* were also observed (Table S2F; Fig. 2D). Of note, these differentially abundant bacterial genera remained significantly associated with rhinitis even after controlling for birth mode, probiotic treatment, antibiotic use, and exclusive breastfeeding in the linear model implemented by *MaAsLin*2 (all *q* < 0.05).

### Gut microbial and early-life factors associated with allergic rhinitis during childhood

**FIG 3:**
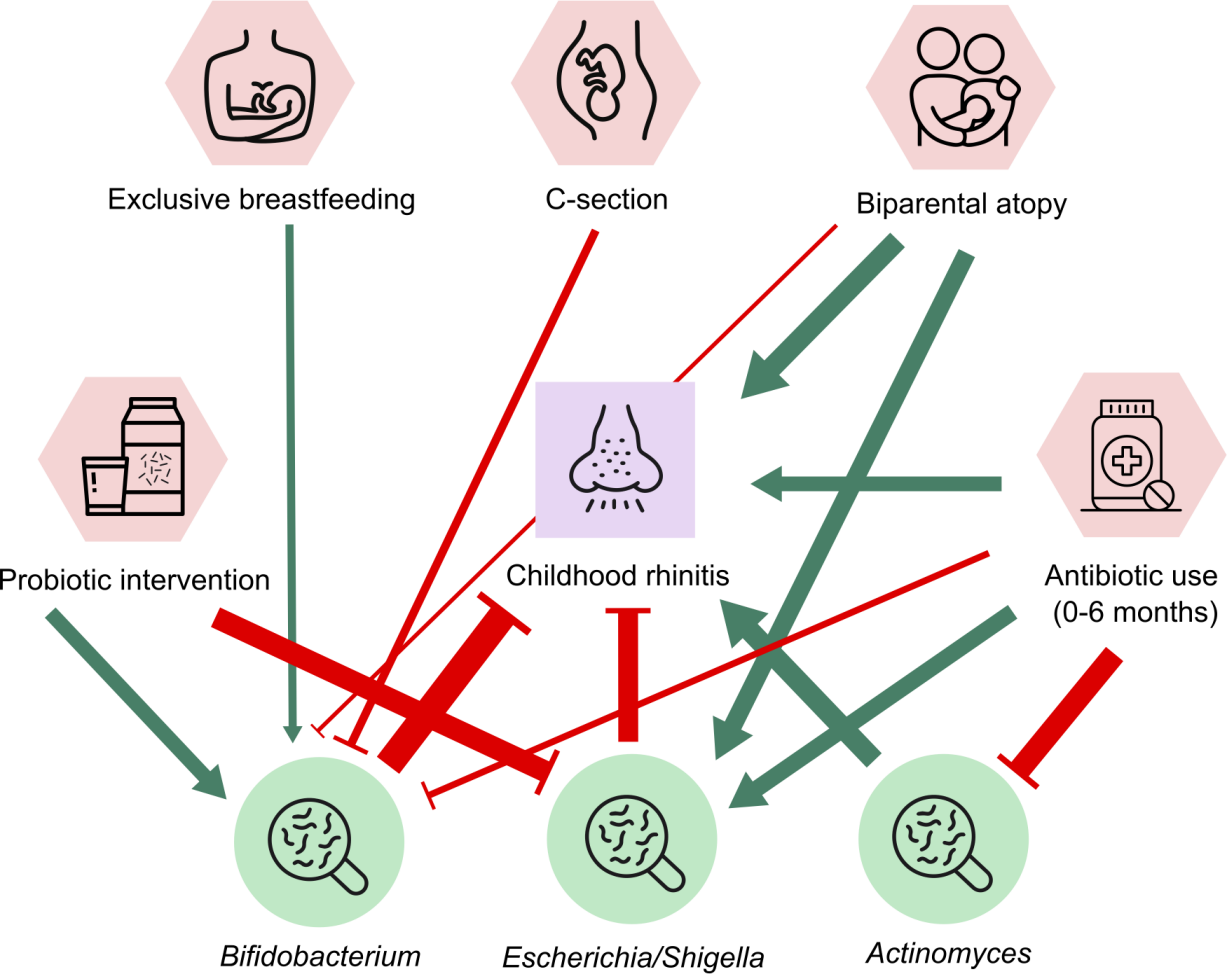
Association network of allergic rhinitis, relative abundances of gut bacteria, and the significant early-life factors identified in the present study (shown in Fig. 1). The parsimonious model was selected based on AIC. Green and red colors indicate positive and negative associations, respectively, and line width is proportional to the model estimate.

We assessed the hypothesis that differences in the gut microbiota and early-life factors as well as their interactions contribute to allergic rhinitis by analyzing the association network. Rhinitis diagnosis was modeled as the outcome variable, while the relative abundance of bacterial genera and the early-life factors that were significantly associated with microbiota composition (see Fig. 1) are shown as the explanatory variables. The multivariate model was generated via stepwise model reduction using the combinations of the explanatory variables, finally arriving at the best model based on Akaike information criterion (AIC). We previously applied this approach to understand the potential effects of intrapartum antibiotics and birth mode on gastrointestinal symptoms mediated by the gut microbiota in infants (36). In the parsimonious model, rhinitis was negatively associated with the relative abundances of *Bifidobacterium* and *Escherichia/Shigella* and positively associated with the relative abundance of *Actinomyces* and biparental atopy, with *Bifidobacterium* having the strongest association strength (Fig. 3). Caesarean section, biparental atopy status, and antibiotic use appeared to reduced *Bifidobacterium*, while exclusive breastfeeding and probiotic treatment promoted its proportion in the gut microbiota (Fig. 3). Biparental atopy status and antibiotic use were associated with an elevated proportion of *Escherichia/Shigella*, though the both early-life factors also directly correlated with rhinitis via this multivariate modeling (Fig. 3). All in all, the association network generated here largely recapitulated the main findings in our cohort as well as those highlighted by previous studies (37).

## DISCUSSION

The overall gut microbiota of the 383 infants in the FLORA intervention cohort at 3 months was associated with birth mode, biparental atopy, exclusive breastfeeding, and antibiotic use. As reported previously (26), the microbiota composition was also strongly associated with the treatment product consisting of a mixture of *Lactobacillus*, *Bifidobacterium*, and *Propionibacterium* spp. complemented with oligosaccharides. The FLORA intervention was successful in altering the gut microbiota, at least in the short term (26). Of note, the impact of the probiotic treatment on the gut microbiota was not limited to the administered microorganisms but also on the overall composition (26). Similar findings have been reported earlier, yet the results are somewhat inconsistent, possibly partly due to differences in treatment protocols and microbiota analysis methods (38).

We have previously shown that the treatment product had a protective effect on allergic morbidity in the entire FLORA cohort, where eczema was less common in the intervention group at 2 years with a relative risk reduction of 26% (17). The decrease in eczema prevalence was also observed afterward, up to 13 years for the cesarean-delivered group, but not in the entire cohort. Moreover, the intervention reduced the potentially detrimental effect of cesarean delivery on the infant’s gut microbiota composition at 3 months (26). Thus, it was unexpected that the only allergic disease significantly associated with microbiota variation was rhinitis, but not eczema. Even in the cesarean section-delivered group, there were no significant microbiota differences associated with other allergic diseases than rhinitis. It is known that the coexistence of allergic disorders is frequent, especially with the presence of certain filaggrin gene variants (39). In our cohort, the prevalence of eczema and other allergic diseases was higher in the rhinitis group compared to others. Robust diagnosis of allergic rhinitis requires positive aeroallergen sensitization testing, for example, serum IgE measurement, which was available only for 174/383 (45.4%) participants. In order to maximize the statistical power, we opted for using rhinitis diagnosis that was based only on self-report of doctor-diagnosed allergic rhinitis. Nevertheless, we performed the additional analysis based on the serum IgE test and confirmed the observed association between the gut microbiota and rhinitis in the participants with aero-IgE testing (Fig. S3).

Remarkably, we found that the strongest association between the gut microbiota and rhinitis was among the children with only one atopic parent, and the status of biparental atopy was significantly associated to infants’ gut microbiota variation. It is plausible that the genetic factors eclipsed host-microbiota interactions, if any, in those infants with biparental atopy as they had a stronger genetic predisposition for allergic disease. This hypothesis may provide some explanation as to why only weak or null associations between the gut microbiota and allergic diseases were observed in our cohort that had a high genetic risk for allergic disease (i.e., with at least one atopic parent). On a broader scale, the findings from other cohorts or populations may not be generalized to the Finnish population. It is worth noting that a recent study on the skin and nasal epithelium microbiota in genetically similar populations with allergy disparities argued that environmental factors exert a larger impact on genetics (40). We cannot rule out the possibility that there are certain unmeasured factors in the families’ living environment, which contribute to allergy development in both parents and children in the same family but had no detectable impact on the gut microbiota.

To our knowledge, this is the first study in the pediatric population reporting associations between the gut microbiota and allergic rhinitis. We found that the relative abundances of *Bifidobacterium* and *Escherichia/Shigella* were negatively associated with rhinitis and that of *Bacteroides* positively associated in the infants with one atopic parent, that is non-biparental atopy. A recent study reported that adults with rhinitis were found to have reduced relative abundances of *Blautia*, *Collinsella*, *Subdoligranulum*, and *Fusinibacter* in the gut microbiota and lower levels of fecal Short-chain fatty acids (SCFAs) compared to healthy controls (41). In another study, adults suffering from allergic rhinitis had higher relative abundances of *Escherichia/Shigella*, *Prevotella*, and *Parabacteroidetes* compared to their healthy counterparts (42). However, as these studies relate to the gut microbiota of adults, it is difficult to compare these with our present findings.

*Bifidobacterium* spp. predominate in the gut microbiota of breastfed infants and have an important role in shaping the early immune system. Prematurity, cesarean delivery, and early antibiotics use have been associated with the reduced abundance of infant-type bifidobacteria, as also corroborated in this study. Early-life antibiotic use is well-known for disrupting gut microbiota homeostasis and its recurrent exposure has been associated with an increased risk of childhood-onset allergic diseases (43). In the present study, antibiotic use was positively associated with childhood rhinitis in the multivariate model (Fig. 3), likely via modulation of the relative abundance of *Bifidobacterium*. Breastfeeding has been reported to reduce the risk of asthma linked to early-life antibiotic use, potentially mediated by *Bifidobacterium longum* subsp. *infantis (*44). The treatment product used in this cohort, containing among others *B. breve* Bb99 (DSM 13692; 2 × 10^8^ CFU per day), was administrated prenatally to the mothers and postnatally to their infants until the age of 6 months. In a murine model of rhinitis, oral administration of *B. breve* reduced the symptoms and serum specific-IgE, IL-4, IL-10, and increased CD4+CD25+ T-regulators (45). In another mouse study using *B. longum* IM55, the similar reduction in symptoms and changes in immunologic parameters were documented (46). Therefore, the negative association between the bifidobacteria and rhinitis observed in the present study is in line with the previous animal studies.

*Bacteroides* species are prevalent in infants delivered vaginally (47). Bacteria belonging to the *Bacteroides* genus have a wide range of effects on human health, ranging from anaerobic infections to anti-inflammatory effects (48). Their role in infants is understandably equivocal, partly due to the heterogenous nature of the genus (48, 49). Hence, it is hard to link this finding to specific properties of particular *Bacteroides* spp. as this would require deep metagenomic analysis. The bacteria belonging to the genus *Escherichia/Shigella* not only are commensals in the human gut but also include opportunistic pathogens. Colonizing adults with a specific orally administered *Escherichia* strain has proven difficult (50), but the colonization in infants is relatively easily achieved (51). This intimate relationship between the human host and the early colonizer’s *Escherichia* spp. highlights the importance of perinatal and early-life microbial exposure that may provide immune support (52, 53), which is in agreement with our findings. The relationship between biparental atopy and the relative abundance of *Escherichia/Shigella* remains to be investigated.

*Actinomyces*, commonly residing in the nasopharynx but also part of the normal gut microbiota, was associated with rhinitis in the association network (Fig. 3). Some species of *Actinomyces* are considered opportunistic pathogens and have been associated with premature delivery (54). Moreover, exposure to particle pollution from fine particulates (PM2.5) at 6 months was positively associated with *Actinomyces* in the gut microbiota (55). The knowledge about the role of intestinal *Actinomyces* and their connections to the immune system is currently limited, but the presence of *Actinomyces* in the upper airway histopathological samples has been associated with allergic rhinitis (56). A specific configuration of the early-life airway microbiota that can be manipulated by antibiotics has been linked to a higher risk of atopy at 5 years of age, though its causality remains debated (35, 57). Importantly, the community structure and particular microbial taxa in the infant gut and airway microbiota were found to be dynamically associated (58), and an altered airway microbiota related to childhood allergic rhinitis and asthma was reflected in the gut microbiota (59). Therefore, there appears to be a host-wide systemic mechanism coordinating the colonization of the early-life microbiota across body sites, which may shed light on the relationship between the microbiota and atopy.

Our study has a few limitations. The sub-cohort from the FLORA intervention analyzed herein represents only a part of the whole cohort (41%), and thus it is possible that the sample size was underpowered. Moreover, more in-depth microbiota profiling methods, such as shotgun metagenomic sequencing and metabolomics, may have provided a more comprehensive landscape of the gut microbiota in this unique cohort. Whether the allergy-associated microbial species and/or metabolites identified from the FLORA and other well-characterized (60) early-life cohorts promote or reflect adaptive immune dysfunction will be our next focus.

In conclusion, our study suggests a connection between infants’ gut microbiota and the occurrence of allergic rhinitis during childhood. On the other hand, the previously reported impact of the probiotic intervention on eczema was not directly associated with the early-life gut microbiota composition. Further investigations building on our findings may help unravel the complex connection among probiotics, gut microbiota, and allergic morbidity.

## Data Availability

The datasets generated in this study are available in the European Nucleotide Archive (ENA) repository under accession PRJEB70729.

## SUPPLEMENTAL MATERIAL

Supplemental figures and tables spectrum.04135-23-s0001.docx Fig. S1-S3; Tables S1-S3.

ASM does not own the copyrights to Supplemental Material that may be linked to, or accessed through, an article. The authors have granted ASM a non-exclusive, world-wide license to publish the Supplemental Material files. Please contact the corresponding author directly for reuse.
