# Supplementary material for: Early-life gut microbiota associates with allergic rhinitis during 13-year follow-up in a Finnish probiotic intervention cohort
Source: Microbiol Spectr. 2024 Apr 30;12(6):e04135-23. doi: 10.1128/spectrum.04135-23 (PMC11324021; doi:10.1128/spectrum.04135-23)
Supplement: Supplemental figures and tables — Fig. S1-S3; Tables S1-S3. [file spectrum.04135-23-s0001.docx]

**Supplementary Information**

**Early-life gut microbiota associates with allergic rhinitis during 13-year follow-up in a Finnish probiotic intervention cohort**

|  | Randomisation of mothers n=1223 |  |
| --- | --- | --- |
| ↓ |  | ↓ |
| Probiotic group mothers 610 |  | Placebo group mothers 613 |
| ↓ |  | ↓ |
| Baseline data available 485  → fecal sample 180 |  | Baseline data available 487  → fecal sample 203 |
| ↓ |  | ↓ |
| 2-year follow-up 461 |  | 2-year follow-up 464 |
| ↓ |  | ↓ |
| 5-year follow-up 445 |  | 5-year follow-up 446 |
| ↓ |  | ↓ |
| 10-year follow-up 407 |  | 10-year follow-up 400 |
| ↓ |  | ↓ |
| 13-year follow-up 330 |  | 13-year follow-up 312 |

**Figure S1.** The flowchart of participants in the probiotic and placebo groups.


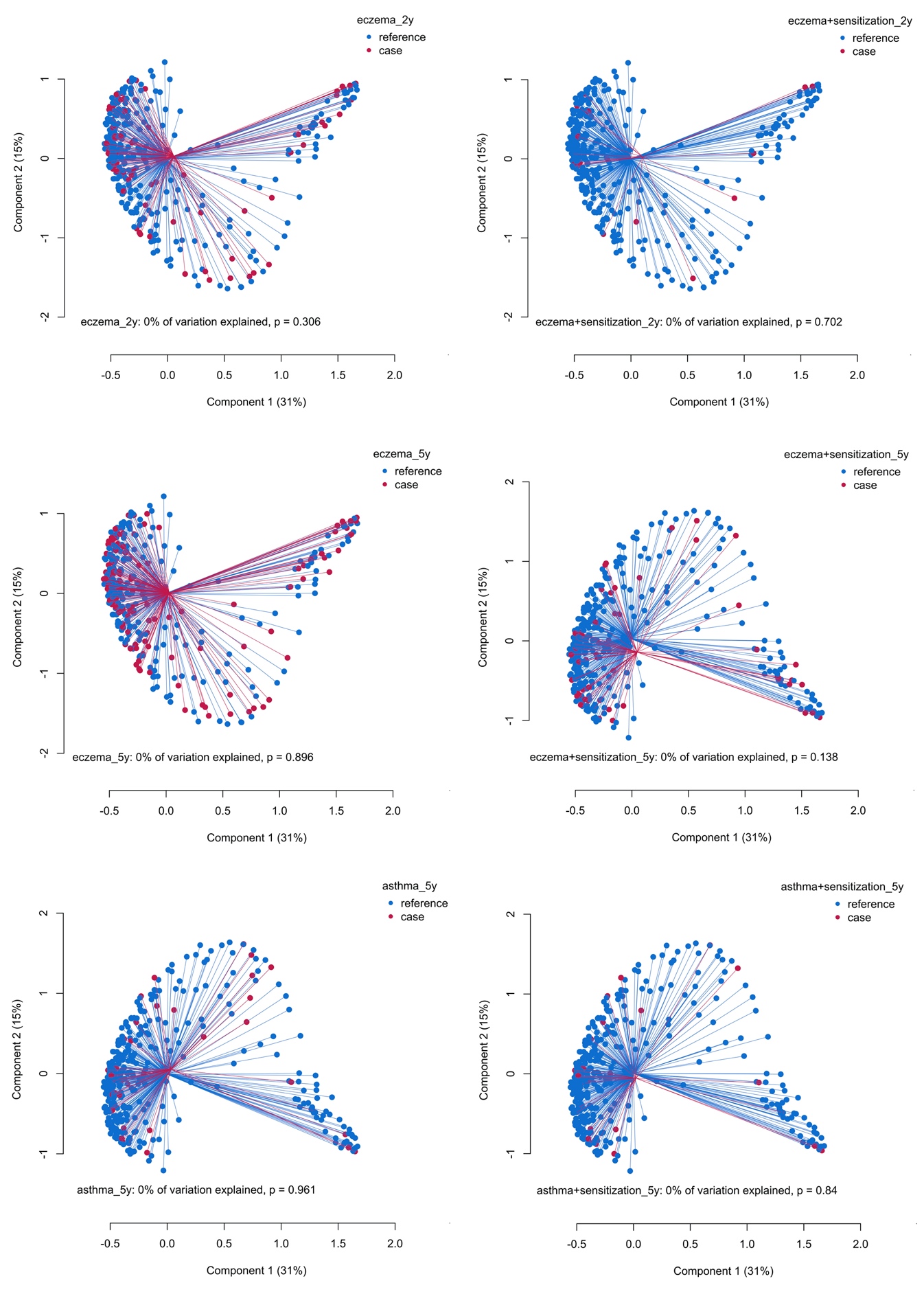


**Figure S2.** Comparison of microbiota β-diversity visualized by the principal coordinate analysis (PCoA) plot between children with and without the diagnosis of eczema or asthma at 2 years (for eczema) and 5 years (for eczema and asthma).


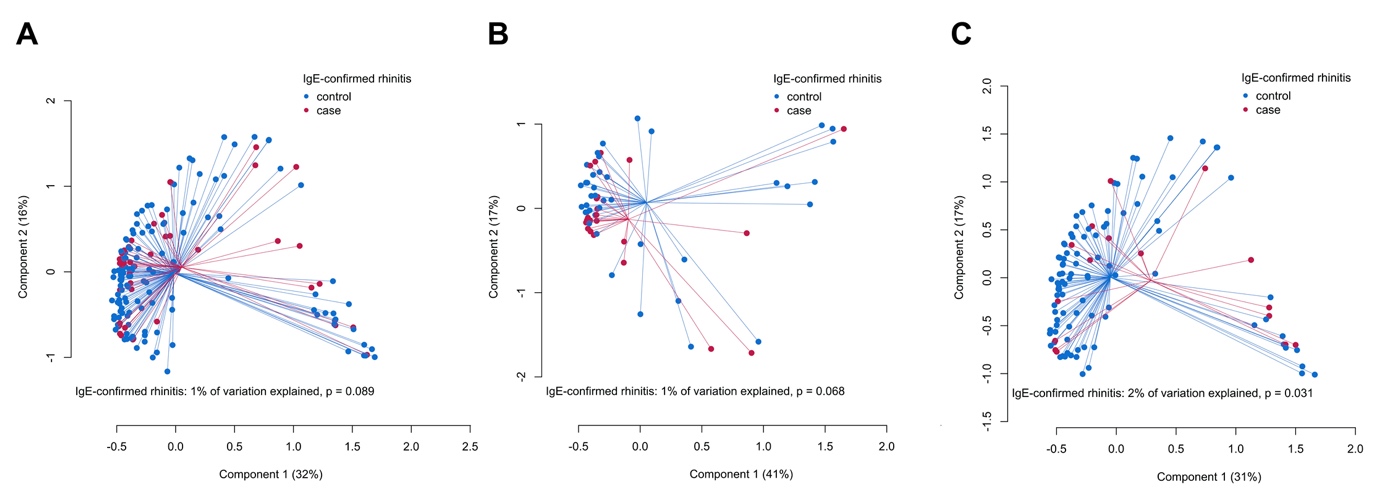


**Figure S3.** Comparison of microbiota β-diversity visualized by the principal coordinate analysis (PCoA) plot between children with IgE-confirmed rhinitis and non-rhinitis controls in the **(A)** overall group (N=174; case/control=38/136), **(B)** biparental atopy (N=63; case/control=22/41) and **(C)** non-biparental atopy (N=111; case/control=16/95) sub-group.

**Table S1.** Comparison of children with and without rhinitis in the present study. * *p*<0.05.

|  | **Rhinitis** | **Control** | ***p*-value** |
| --- | --- | --- | --- |
|  | n=99 | n=174 |  |
| Treatment group (%) | 51.5 | 54.6 | 0.7153 |
| Female (%) | 44.4 | 55.2 | 0.1143 |
| Birth weight (g) | 3528 | 3522 | 0.9172 |
| Birth height (cm) | 50.3 | 50.3 | 0.9992 |
| Mothers’ age at labor (y/o) | 31.31 | 31.25 | 0.9310 |
| BMI at 5y | 15.9 | 15.7 | 0.4855 |
| Maternal atopy (%) | 82.8 | 81 | 0.8371 |
| Paternal atopy (%) | 64.6 | 52.9 | 0.078 |
| Biparental atopy (%) | 47.5 | 33.9 | 0.0373* |
| Caesarean birth (%) | 14.1 | 9.8 | 0.3702 |
| Fully breastfed at 3m (%) | 43.4 | 53.4 | 0.1429 |
| Fully breastfed at 5m (%) | 7.1 | 3.4 | 0.2911 |
| Breastfed over 6m (%) | 61.6 | 64.4 | 0.7467 |
| Smokers at home 0-2 y/o (%) | 34.3 | 33.3 | 0.9708 |
| Dogs/cats at home 0-2 y/o (%) | 19.2 | 20.1 | 0.9792 |
| Parents with higher education (%) | 44.4 | 48.3 | 0.6286 |
| Antibiotics during intervention (%) | 20.2 | 20.7 | 0.7770 |
| Eczema ever (%) | 58.6 | 41.4 | 0.0003* |
| Asthma ever (%) | 27.3 | 9.2 | <0.0001* |
| Food allergy ever (%) | 54.5 | 13.8 | <0.0001* |
| Any allergic disease ever (%) | 100 | 53.4 | <0.0001* |
| Siblings >1 (%) | 43.4 | 41.4 | 0.74094 |
| Infections 0-2 y/o (%) | 93.9 | 92.5 | 0.8470 |
| Infections 3-5 y/o (%) | 100 | 99.4 | 1.0000 |

**Table S2.** Differences in individual bacterial taxa between those with and without allergic disease (any allergy, asthma, eczema, food allergy, allergic rhinitis and allergic rhinitis in non-biparental atopy group)

A. Any allergy ever

| **feature** | **coef** | **stderr** | **q (adjusted p)** |
| --- | --- | --- | --- |
| Clostridium_sensu_stricto_1 | -0.8466532 | 0.29847865 | 0.06118188 |
| Collinsella | 0.83800806 | 0.37098239 | 0.20728639 |
| Escherichia.Shigella | -0.9167222 | 0.41478228 | 0.21546568 |
| Ruminiclostridium_5 | 0.27204936 | 0.13692787 | 0.30249429 |
| Enterococcus | -0.575787 | 0.30314175 | 0.35043834 |
| Klebsiella | -0.5920609 | 0.34765707 | 0.42104927 |
| Gemella | 0.19682727 | 0.14391041 | 0.57693951 |
| Bacteroides | 0.50218085 | 0.38562585 | 0.59907138 |
| Granulicatella | 0.16726145 | 0.13836274 | 0.59916936 |
| Anaerococcus | -0.1543007 | 0.12654413 | 0.59916936 |
| Akkermansia | 0.20159826 | 0.16686467 | 0.59916936 |
| Clostridioides | -0.2317784 | 0.21670272 | 0.67254669 |
| Pseudomonas | 0.09332325 | 0.09453627 | 0.71323926 |
| Peptoniphilus | 0.15778223 | 0.16457957 | 0.72140909 |
| Sphingomonas | 0.18778528 | 0.20573218 | 0.75087324 |
| Negativicoccus | 0.11471913 | 0.12664752 | 0.75186086 |
| Haemophilus | 0.21759629 | 0.2520529 | 0.76777197 |
| Blautia | 0.24387505 | 0.29317947 | 0.77831989 |
| Tyzzerella_4 | 0.16632221 | 0.19795801 | 0.77831989 |
| Burkholderia.Caballeronia.Paraburkholderia | 0.09755062 | 0.1281602 | 0.78052039 |
| Halomonas | 0.0676581 | 0.09879396 | 0.78942212 |
| Corynebacterium_1 | -0.0783468 | 0.1332136 | 0.8310258 |
| Finegoldia | -0.0581272 | 0.10072204 | 0.8310258 |
| Veillonella | -0.1902658 | 0.32744378 | 0.8310258 |
| Rothia | 0.12460606 | 0.21773411 | 0.83279295 |
| Streptococcus | 0.10119182 | 0.1794051 | 0.83328702 |
| Lactobacillus | -0.1469168 | 0.31317746 | 0.87495405 |
| Parabacteroides | 0.09395226 | 0.23507534 | 0.90930414 |
| Actinomyces | 0.10294757 | 0.26935547 | 0.91045537 |
| Enterobacter | -0.0761301 | 0.20752621 | 0.91334318 |
| Achromobacter | 0.03429484 | 0.10189921 | 0.91929102 |
| Bifidobacterium | -0.0542393 | 0.26793439 | 0.93526326 |
| Staphylococcus | -0.0517389 | 0.21016379 | 0.93526326 |
| Subdoligranulum | 0.04391875 | 0.16230786 | 0.93526326 |
| Variovorax | 0.01414666 | 0.11696207 | 0.96073902 |
| Flavonifractor | -0.0220398 | 0.19926508 | 0.96438519 |
| Curvibacter | -0.0088332 | 0.13269884 | 0.98451273 |
| Erysipelatoclostridium | -0.0139509 | 0.25186581 | 0.99008766 |
| Eggerthella | 0.0063933 | 0.31593814 | 0.99367104 |
| Pseudobutyrivibrio | -0.0020794 | 0.12109334 | 0.99367104 |
| Faecalibacterium | 0.00310218 | 0.11921964 | 0.99367104 |
| Atopobium | 0.00137426 | 0.13720162 | 0.99446999 |

B. Asthma ever

| **feature** | **coef** | **stderr** | **q (adjusted p)** |
| --- | --- | --- | --- |
| Lactobacillus | -0.6327195 | 0.3595316 | 0.39451233 |
| Ruminiclostridium_5 | 0.27196743 | 0.16234567 | 0.42174096 |
| Enterococcus | -0.4819053 | 0.33941104 | 0.57031839 |
| Burkholderia.Caballeronia.Paraburkholderia | 0.17561586 | 0.14370562 | 0.62984332 |
| Sphingomonas | 0.27516855 | 0.23397424 | 0.63695897 |
| Rothia | -0.2757524 | 0.25219549 | 0.67860417 |
| Actinomyces | -0.3177358 | 0.30491169 | 0.71601098 |
| Bacteroides | 0.45526104 | 0.45134039 | 0.72656052 |
| Enterobacter | 0.23618133 | 0.23408897 | 0.72656052 |
| Tyzzerella_4 | -0.201433 | 0.20937978 | 0.7492373 |
| Parabacteroides | 0.22812898 | 0.27155354 | 0.81337557 |
| Clostridioides | 0.18313469 | 0.24229261 | 0.83537593 |
| Blautia | -0.2318222 | 0.33273573 | 0.84624983 |
| Curvibacter | 0.10011449 | 0.15545647 | 0.87909694 |
| Peptoniphilus | 0.11954331 | 0.18960204 | 0.88976453 |
| Bifidobacterium | -0.1601157 | 0.30971496 | 0.89885013 |
| Corynebacterium_1 | 0.07774119 | 0.15579568 | 0.89885013 |
| Collinsella | 0.25212248 | 0.423789 | 0.89885013 |
| Eggerthella | -0.1529347 | 0.35439005 | 0.89885013 |
| Staphylococcus | -0.1290784 | 0.24740725 | 0.89885013 |
| Granulicatella | 0.07202215 | 0.16183476 | 0.89885013 |
| Subdoligranulum | -0.0940551 | 0.18298111 | 0.89885013 |
| Achromobacter | 0.06475721 | 0.1144942 | 0.89885013 |
| Haemophilus | -0.133205 | 0.29148693 | 0.89885013 |
| Negativicoccus | -0.0599616 | 0.14852799 | 0.90561239 |
| Atopobium | -0.0455828 | 0.15465734 | 0.94270256 |
| Faecalibacterium | 0.03395668 | 0.13294515 | 0.94270256 |
| Variovorax | -0.0253741 | 0.13100251 | 0.94270256 |
| Escherichia.Shigella | 0.08763293 | 0.4716427 | 0.94270256 |
| Klebsiella | 0.11533856 | 0.40644514 | 0.94270256 |
| Halomonas | -0.0291456 | 0.11537122 | 0.94270256 |
| Veillonella | -0.0390026 | 0.3787757 | 0.98378857 |
| Akkermansia | -0.0153018 | 0.17880406 | 0.98378857 |
| Gemella | -0.0098814 | 0.16624504 | 0.98833739 |
| Erysipelatoclostridium | -0.0187161 | 0.27911117 | 0.98833739 |
| Pseudomonas | -0.0070654 | 0.1093936 | 0.98833739 |
| Pseudobutyrivibrio | 0.00769091 | 0.14112944 | 0.98871168 |
| Flavonifractor | 0.00709195 | 0.22617819 | 0.99557231 |
| Clostridium_sensu_stricto_1 | 0.00865764 | 0.34782831 | 0.99562356 |
| Anaerococcus | 0.00304696 | 0.14080193 | 0.99562356 |
| Streptococcus | 0.0005138 | 0.20402784 | 0.99938153 |
| Finegoldia | -0.0010519 | 0.11364086 | 0.99938153 |

C. Eczema ever

| **feature** | **coef** | **stderr** | **q (adjusted p)** |
| --- | --- | --- | --- |
| Atopobium | -0.2610637 | 0.12009296 | 0.24225567 |
| Escherichia.Shigella | -0.8126762 | 0.37741172 | 0.24605 |
| Clostridium_sensu_stricto_1 | -0.5305944 | 0.27271214 | 0.31821848 |
| Haemophilus | 0.38766481 | 0.2317013 | 0.45975199 |
| Akkermansia | 0.24628319 | 0.15311708 | 0.47098396 |
| Klebsiella | -0.4807551 | 0.31023133 | 0.50536753 |
| Blautia | 0.40146071 | 0.26742275 | 0.51935187 |
| Finegoldia | -0.1311471 | 0.09118212 | 0.53477194 |
| Clostridioides | -0.2687108 | 0.19457377 | 0.55419195 |
| Bacteroides | 0.41980946 | 0.35495978 | 0.6508289 |
| Anaerococcus | -0.1000322 | 0.10819219 | 0.7760694 |
| Collinsella | 0.26335597 | 0.33276924 | 0.80500873 |
| Burkholderia.Caballeronia.Paraburkholderia | 0.08949834 | 0.11301387 | 0.80500873 |
| Subdoligranulum | 0.1121896 | 0.14888758 | 0.81053289 |
| Enterobacter | 0.13940575 | 0.19001473 | 0.81053289 |
| Halomonas | -0.063203 | 0.08913829 | 0.81406831 |
| Negativicoccus | 0.08075448 | 0.11599996 | 0.81480871 |
| Peptoniphilus | 0.09092258 | 0.14681769 | 0.84954033 |
| Rothia | 0.11975603 | 0.19496171 | 0.8502127 |
| Staphylococcus | 0.10890706 | 0.18703042 | 0.86655834 |
| Sphingomonas | 0.10056556 | 0.18543608 | 0.87067732 |
| Tyzzerella_4 | 0.08781098 | 0.17768013 | 0.87401875 |
| Pseudomonas | -0.0373989 | 0.08612857 | 0.90294486 |
| Gemella | -0.0528438 | 0.1305613 | 0.90492784 |
| Ruminiclostridium_5 | 0.04906646 | 0.12501698 | 0.90689211 |
| Curvibacter | -0.047123 | 0.12127829 | 0.90689211 |
| Veillonella | -0.0996343 | 0.294693 | 0.92800546 |
| Bifidobacterium | 0.08024185 | 0.24290269 | 0.9295837 |
| Achromobacter | 0.0301221 | 0.09095763 | 0.9295837 |
| Enterococcus | -0.0855937 | 0.27080535 | 0.93019788 |
| Corynebacterium_1 | -0.0367396 | 0.12066631 | 0.93393103 |
| Granulicatella | 0.02920781 | 0.12625528 | 0.95463367 |
| Flavonifractor | -0.0394862 | 0.17938934 | 0.95463367 |
| Eggerthella | 0.0301831 | 0.28590501 | 0.98347491 |
| Parabacteroides | -0.0220451 | 0.20989321 | 0.98347491 |
| Lactobacillus | 0.03268723 | 0.28629994 | 0.98347491 |
| Faecalibacterium | -0.008103 | 0.10714079 | 0.98347491 |
| Erysipelatoclostridium | 0.02878069 | 0.22922009 | 0.98347491 |
| Actinomyces | 0.01497291 | 0.24701335 | 0.98520063 |
| Pseudobutyrivibrio | 0.00685584 | 0.10972964 | 0.98520063 |
| Variovorax | -0.0059787 | 0.10261288 | 0.98520063 |
| Streptococcus | 0.00604218 | 0.16162214 | 0.98637549 |

D. Food allergy ever

| **feature** | **coef** | **stderr** | **q (adjusted p)** |
| --- | --- | --- | --- |
| Tyzzerella_4 | 0.42415055 | 0.1996419 | 0.28534005 |
| Ruminiclostridium_5 | 0.24505681 | 0.14477301 | 0.5087321 |
| Pseudobutyrivibrio | -0.1976949 | 0.12803594 | 0.58290222 |
| Collinsella | 0.52282169 | 0.38423487 | 0.62991644 |
| Actinomyces | 0.34647039 | 0.27649032 | 0.67600054 |
| Clostridium_sensu_stricto_1 | -0.3445979 | 0.30246659 | 0.71892505 |
| Faecalibacterium | -0.136855 | 0.11912487 | 0.71892505 |
| Haemophilus | -0.3001702 | 0.26269355 | 0.71892505 |
| Akkermansia | 0.1473866 | 0.16716996 | 0.81692819 |
| Atopobium | 0.11921337 | 0.14080297 | 0.83123696 |
| Sphingomonas | 0.17693072 | 0.21278248 | 0.83123696 |
| Burkholderia.Caballeronia.Paraburkholderia | -0.100636 | 0.1258857 | 0.83917221 |
| Corynebacterium_1 | -0.105978 | 0.13978604 | 0.84272622 |
| Staphylococcus | -0.1606027 | 0.21902599 | 0.84348271 |
| Erysipelatoclostridium | -0.1893116 | 0.25753809 | 0.84348271 |
| Escherichia.Shigella | -0.3002488 | 0.42226437 | 0.84481708 |
| Rothia | 0.1583785 | 0.22738373 | 0.85425532 |
| Veillonella | -0.2220589 | 0.34110917 | 0.87926693 |
| Parabacteroides | -0.1241002 | 0.23731598 | 0.90275972 |
| Streptococcus | 0.10545839 | 0.18403639 | 0.90275972 |
| Blautia | 0.15910878 | 0.30098766 | 0.90275972 |
| Curvibacter | -0.0713791 | 0.13810716 | 0.90520191 |
| Granulicatella | 0.07360348 | 0.14644618 | 0.90580513 |
| Anaerococcus | -0.0600868 | 0.12444716 | 0.90580513 |
| Negativicoccus | 0.06687999 | 0.13121153 | 0.90580513 |
| Variovorax | -0.055254 | 0.12223647 | 0.90580513 |
| Klebsiella | -0.165861 | 0.35558091 | 0.90580513 |
| Halomonas | 0.05010079 | 0.10409149 | 0.90580513 |
| Pseudomonas | 0.04889293 | 0.09755801 | 0.90580513 |
| Eggerthella | 0.1245456 | 0.32181428 | 0.92475181 |
| Flavonifractor | -0.068178 | 0.19616768 | 0.93261121 |
| Clostridioides | -0.0646289 | 0.2158478 | 0.94729983 |
| Gemella | 0.04235499 | 0.15246953 | 0.95312114 |
| Lactobacillus | 0.07574212 | 0.32164678 | 0.95312114 |
| Finegoldia | -0.0278299 | 0.100982 | 0.95312114 |
| Achromobacter | 0.02220004 | 0.10385921 | 0.95312114 |
| Subdoligranulum | -0.029302 | 0.15872473 | 0.96038756 |
| Enterococcus | 0.05475766 | 0.31640287 | 0.96084511 |
| Bifidobacterium | 0.02762817 | 0.26985875 | 0.97006094 |
| Enterobacter | 0.02329241 | 0.20204862 | 0.97006094 |
| Bacteroides | 0.02349966 | 0.40973643 | 0.98474897 |
| Peptoniphilus | 0.00479642 | 0.16995049 | 0.98474897 |

E. Rhinitis ever

| **feature** | **coef** | **stderr** | **q (adjusted p)** |
| --- | --- | --- | --- |
| Escherichia.Shigella | -0.4003964 | 0.15375749 | 0.04656921 |
| Bifidobacterium | -0.9465069 | 0.40113279 | 0.17123827 |
| Actinomyces | -0.591256 | 0.25954413 | 0.20671907 |
| Pseudomonas | 0.18019207 | 0.09386363 | 0.35983786 |
| Bacteroides | 0.72998621 | 0.39509456 | 0.39963916 |
| Burkholderia.Caballeronia.Paraburkholderia | -0.212499 | 0.11933675 | 0.42102385 |
| Halomonas | 0.17834561 | 0.10111717 | 0.42102385 |
| Peptoniphilus | 0.28244003 | 0.17043847 | 0.44906763 |
| Corynebacterium_1 | -0.2071137 | 0.13622967 | 0.49785061 |
| Sphingomonas | -0.2905788 | 0.20335571 | 0.55011168 |
| Ruminiclostridium_5 | 0.18374029 | 0.14155992 | 0.61686693 |
| Staphylococcus | -0.2601346 | 0.21288047 | 0.65390239 |
| Curvibacter | 0.15300659 | 0.1331517 | 0.67469412 |
| Akkermansia | 0.16164842 | 0.15824663 | 0.72778586 |
| Finegoldia | 0.0863571 | 0.09876526 | 0.77114427 |
| Faecalibacterium | 0.09619887 | 0.11561565 | 0.77393466 |
| Flavonifractor | -0.1587283 | 0.19103412 | 0.77393466 |
| Atopobium | -0.1016781 | 0.13951728 | 0.78060052 |
| Parabacteroides | 0.15325511 | 0.23350304 | 0.81790415 |
| Rothia | 0.10937788 | 0.22349181 | 0.89232127 |
| Subdoligranulum | 0.06348076 | 0.15450523 | 0.92932672 |
| Streptococcus | -0.0709957 | 0.17532953 | 0.93212559 |
| Klebsiella | -0.1140595 | 0.34576123 | 0.97241846 |
| Granulicatella | 0.03862996 | 0.14315242 | 0.97336726 |
| Veillonella | 0.10042059 | 0.33429518 | 0.97336726 |
| Enterobacter | -0.0508097 | 0.19346763 | 0.97425325 |
| Gemella | 0.03019168 | 0.1484572 | 0.97675859 |
| Clostridium_sensu_stricto_1 | 0.06208766 | 0.30133783 | 0.97675859 |
| Erysipelatoclostridium | 0.05508927 | 0.25411004 | 0.97675859 |
| Enterococcus | -0.0415071 | 0.30783762 | 0.97793893 |
| Lactobacillus | -0.0587255 | 0.31155432 | 0.97793893 |
| Pseudobutyrivibrio | 0.01854869 | 0.12564264 | 0.97793893 |
| Clostridioides | -0.0276291 | 0.21230906 | 0.97793893 |
| Negativicoccus | -0.0198034 | 0.13087465 | 0.97793893 |
| Variovorax | 0.01467843 | 0.11958918 | 0.97793893 |
| Haemophilus | 0.04411026 | 0.25967939 | 0.97793893 |
| Collinsella | 0.02277924 | 0.3727928 | 0.99238475 |
| Eggerthella | -0.0170933 | 0.31219637 | 0.99238475 |
| Anaerococcus | 0.00776374 | 0.13226297 | 0.99238475 |
| Blautia | -0.0037122 | 0.29221384 | 0.99238475 |
| Tyzzerella_4 | 0.00646099 | 0.17938449 | 0.99238475 |
| Achromobacter | -0.0047081 | 0.10149604 | 0.99238475 |

F. Rhinitis ever (non-biparental atopy)

| **feature** | **coef** | **stderr** | **q (adjusted p)** |
| --- | --- | --- | --- |
| Bifidobacterium | -0.8933746 | 0.30797344 | 0.00163752 |
| Escherichia.Shigella | -1.3788826 | 0.52758418 | 0.00545847 |
| Bacteroides | 1.18616424 | 0.52618444 | 0.00545847 |
| Burkholderia.Caballeronia.Paraburkholderia | -0.3560801 | 0.17157555 | 0.07171084 |
| Actinomyces | 0.51492626 | 0.35506064 | 0.20154913 |
| Flavonifractor | -0.3368756 | 0.2463534 | 0.21018319 |
| Atopobium | -0.2529922 | 0.1918437 | 0.27411724 |
| Pseudomonas | 0.16557084 | 0.13058828 | 0.2920428 |
| Akkermansia | 0.26724672 | 0.228524 | 0.30920116 |
| Clostridium_sensu_stricto_1 | 0.44607185 | 0.41082756 | 0.33998377 |
| Sphingomonas | -0.296254 | 0.27677156 | 0.37539455 |
| Corynebacterium_1 | -0.1606012 | 0.17524891 | 0.38920803 |
| Collinsella | -0.4713102 | 0.50014535 | 0.46329547 |
| Anaerococcus | -0.1794709 | 0.19845505 | 0.49342399 |
| Pseudobutyrivibrio | 0.15738458 | 0.16636314 | 0.51085332 |
| Clostridioides | -0.2536022 | 0.27282746 | 0.51833697 |
| Negativicoccus | 0.13911007 | 0.1623455 | 0.56191632 |
| Enterococcus | 0.33220776 | 0.39431767 | 0.56562759 |
| Halomonas | 0.11190983 | 0.13742105 | 0.61486726 |
| Staphylococcus | 0.20447314 | 0.27223858 | 0.66585692 |
| Finegoldia | 0.10744029 | 0.13996969 | 0.66585692 |
| Faecalibacterium | 0.11708036 | 0.15613198 | 0.67035276 |
| Veillonella | 0.28815151 | 0.41037946 | 0.67035276 |
| Parabacteroides | 0.20180066 | 0.29971178 | 0.67035276 |
| Peptoniphilus | 0.15585599 | 0.24322627 | 0.67035276 |
| Rothia | 0.17769553 | 0.29338324 | 0.7022733 |
| Subdoligranulum | -0.0984207 | 0.19474407 | 0.7022733 |
| Achromobacter | -0.0704009 | 0.1372536 | 0.75452561 |
| Enterobacter | -0.1336496 | 0.26979622 | 0.75452561 |
| Curvibacter | 0.08271756 | 0.18464204 | 0.75452561 |
| Klebsiella | -0.2180696 | 0.48603465 | 0.79481705 |
| Blautia | 0.1650434 | 0.39365234 | 0.80260119 |
| Haemophilus | 0.14085492 | 0.35074888 | 0.86047986 |
| Variovorax | 0.05930544 | 0.16657141 | 0.87326884 |
| Lactobacillus | 0.11848616 | 0.36693355 | 0.89560399 |
| Streptococcus | -0.0544873 | 0.22856172 | 0.9321363 |
| Ruminiclostridium_5 | -0.0373963 | 0.18843373 | 0.9321363 |
| Granulicatella | -0.024417 | 0.20119107 | 0.96353996 |
| Erysipelatoclostridium | 0.04091443 | 0.2952303 | 0.96353996 |
| Eggerthella | -0.0065487 | 0.41354278 | 0.96353996 |
| Gemella | 0.00334514 | 0.20228399 | 0.99367564 |

**Table S3.** Differences in individual bacterial taxa between those with and without allergic disease (eczema and asthma with and without sensitization at 2 and 5 years)

A. Eczema at 2 years

| **feature** | **coef** | **stderr** | **q (adjusted p)** |
| --- | --- | --- | --- |
| Haemophilus | 0.63494892 | 0.23390776 | 0.07411387 |
| Staphylococcus | 0.53559658 | 0.20339653 | 0.0871087 |
| Clostridium_sensu_stricto_1 | -0.7057994 | 0.28677679 | 0.12882607 |
| Pseudobutyrivibrio | -0.2864505 | 0.11842211 | 0.13549689 |
| Klebsiella | -0.7895055 | 0.33024961 | 0.1432244 |
| Lactobacillus | 0.64408939 | 0.29574585 | 0.20990612 |
| Collinsella | -0.6899743 | 0.35727293 | 0.29286126 |
| Corynebacterium_1 | 0.21860991 | 0.12983234 | 0.40994218 |
| Escherichia.Shigella | -0.6670838 | 0.39514406 | 0.40994218 |
| Bacteroides | 0.56546507 | 0.36993529 | 0.48023351 |
| Pseudomonas | -0.1259174 | 0.09313528 | 0.56074203 |
| Akkermansia | 0.23632927 | 0.17844655 | 0.5620785 |
| Clostridioides | -0.2811683 | 0.21491596 | 0.56519832 |
| Burkholderia.Caballeronia.Paraburkholderia | 0.1581013 | 0.12340593 | 0.56519832 |
| Atopobium | -0.1623882 | 0.12979764 | 0.57878622 |
| Curvibacter | -0.1561204 | 0.12707061 | 0.58146378 |
| Granulicatella | 0.15058256 | 0.13924162 | 0.63913865 |
| Tyzzerella_4 | 0.21323247 | 0.19875763 | 0.63913865 |
| Bifidobacterium | -0.2456774 | 0.25665683 | 0.67651477 |
| Eggerthella | 0.29406907 | 0.30243752 | 0.67651477 |
| Flavonifractor | 0.17757246 | 0.18771396 | 0.68095802 |
| Halomonas | -0.0890225 | 0.09636753 | 0.69358997 |
| Anaerococcus | -0.1116809 | 0.12462919 | 0.70680532 |
| Sphingomonas | 0.14150571 | 0.19970533 | 0.80769204 |
| Rothia | -0.1279821 | 0.21027298 | 0.85144647 |
| Streptococcus | 0.09508864 | 0.17358379 | 0.87950961 |
| Faecalibacterium | -0.0628349 | 0.11458287 | 0.87950961 |
| Subdoligranulum | -0.0803398 | 0.1524523 | 0.88146733 |
| Achromobacter | -0.0529433 | 0.10030771 | 0.88146733 |
| Blautia | 0.14676828 | 0.29200733 | 0.88809255 |
| Negativicoccus | 0.06287027 | 0.13159772 | 0.8886035 |
| Actinomyces | -0.1094179 | 0.26115192 | 0.88876559 |
| Finegoldia | -0.044857 | 0.09751324 | 0.88876559 |
| Ruminiclostridium_5 | -0.0595514 | 0.1335408 | 0.88876559 |
| Enterobacter | 0.07809411 | 0.18631897 | 0.88876559 |
| Parabacteroides | -0.0726763 | 0.22668852 | 0.91057885 |
| Erysipelatoclostridium | 0.07592312 | 0.24830578 | 0.9187566 |
| Enterococcus | 0.0808414 | 0.29287883 | 0.92530056 |
| Peptoniphilus | 0.04042791 | 0.16213903 | 0.92627092 |
| Variovorax | -0.0193776 | 0.11439575 | 0.96308082 |
| Veillonella | -0.0202842 | 0.31300932 | 0.98169028 |
| Gemella | -0.001487 | 0.13777673 | 0.99631501 |

*The participants without the given indication were used as the reference group*

B. Eczema with sensitization at 2 years

| **feature** | **coef** | **stderr** | **q (adjusted p)** |
| --- | --- | --- | --- |
| Tyzzerella_4 | 0.83751345 | 0.40864422 | 0.28724377 |
| Pseudobutyrivibrio | -0.481045 | 0.24507913 | 0.30038613 |
| Collinsella | -1.2130078 | 0.73850136 | 0.4756011 |
| Staphylococcus | 0.63689992 | 0.42184555 | 0.52382794 |
| Eggerthella | 0.855189 | 0.62101248 | 0.54739435 |
| Peptoniphilus | 0.47691217 | 0.33361213 | 0.54739435 |
| Ruminiclostridium_5 | -0.3607304 | 0.27505102 | 0.54739435 |
| Subdoligranulum | 0.41945507 | 0.31406077 | 0.54739435 |
| Sphingomonas | 0.57696819 | 0.4107006 | 0.54739435 |
| Haemophilus | 0.62361499 | 0.48382944 | 0.55758654 |
| Actinomyces | 0.65347125 | 0.53647051 | 0.58553746 |
| Clostridium_sensu_stricto_1 | -0.6585835 | 0.59562902 | 0.6422711 |
| Burkholderia.Caballeronia.Paraburkholderia | 0.2680296 | 0.25456267 | 0.67062975 |
| Enterobacter | -0.3799641 | 0.38411276 | 0.68840698 |
| Klebsiella | -0.6492635 | 0.6861625 | 0.7050011 |
| Enterococcus | 0.54508626 | 0.60242459 | 0.72542159 |
| Lactobacillus | 0.54795228 | 0.6137499 | 0.72542159 |
| Achromobacter | -0.1818312 | 0.20681269 | 0.72963275 |
| Faecalibacterium | -0.1803395 | 0.23637416 | 0.78730911 |
| Pseudomonas | 0.14161534 | 0.1925576 | 0.79164127 |
| Atopobium | -0.1867309 | 0.26648167 | 0.80987398 |
| Rothia | -0.2899015 | 0.433464 | 0.82314501 |
| Parabacteroides | -0.2985822 | 0.46745712 | 0.82481164 |
| Anaerococcus | -0.1577365 | 0.25725227 | 0.83817513 |
| Variovorax | -0.131754 | 0.23595387 | 0.87511126 |
| Bacteroides | -0.4085243 | 0.76211718 | 0.88602047 |
| Gemella | -0.1266542 | 0.28314349 | 0.89315552 |
| Veillonella | -0.2869458 | 0.64495492 | 0.89315552 |
| Curvibacter | -0.1144985 | 0.26275215 | 0.89315552 |
| Corynebacterium_1 | -0.1083784 | 0.26752715 | 0.90584808 |
| Clostridioides | -0.181309 | 0.44454576 | 0.90584808 |
| Akkermansia | -0.1431809 | 0.36911427 | 0.91397534 |
| Erysipelatoclostridium | -0.1796816 | 0.51242343 | 0.91449321 |
| Bifidobacterium | -0.1783722 | 0.53013682 | 0.9215415 |
| Halomonas | 0.06629957 | 0.19910137 | 0.9215415 |
| Blautia | 0.19226711 | 0.60273394 | 0.92989756 |
| Finegoldia | 0.04232779 | 0.20127591 | 0.95917108 |
| Escherichia.Shigella | 0.12806855 | 0.81703509 | 0.97414866 |
| Negativicoccus | 0.03486646 | 0.27165793 | 0.97629273 |
| Granulicatella | -0.0203143 | 0.28695375 | 0.98494517 |
| Flavonifractor | 0.02781966 | 0.38764693 | 0.98494517 |
| Streptococcus | -0.0170131 | 0.35818627 | 0.98627018 |

*The participants without the given indication were used as the reference group*

C. Eczema at 5 years

| **feature** | **coef** | **stderr** | **q (adjusted p)** |
| --- | --- | --- | --- |
| Haemophilus | 0.79822346 | 0.20637679 | 0.00293639 |
| Klebsiella | -0.712909 | 0.2961036 | 0.13899934 |
| Burkholderia.Caballeronia.Paraburkholderia | 0.25504154 | 0.10726343 | 0.14536563 |
| Curvibacter | -0.2618768 | 0.11144075 | 0.15343471 |
| Staphylococcus | 0.41445261 | 0.18173497 | 0.17373701 |
| Pseudomonas | -0.1823796 | 0.08180489 | 0.18438567 |
| Pseudobutyrivibrio | -0.2223392 | 0.10587202 | 0.22694642 |
| Halomonas | -0.1637331 | 0.08551954 | 0.28886511 |
| Escherichia.Shigella | -0.6335616 | 0.35135426 | 0.32490029 |
| Akkermansia | 0.26263122 | 0.15525996 | 0.37856323 |
| Faecalibacterium | -0.1633177 | 0.10138367 | 0.41691041 |
| Clostridium_sensu_stricto_1 | -0.40897 | 0.25634524 | 0.42605402 |
| Parabacteroides | -0.2507041 | 0.20227171 | 0.59839223 |
| Gemella | 0.15109114 | 0.12361534 | 0.60862864 |
| Sphingomonas | 0.20488516 | 0.17731469 | 0.63338693 |
| Anaerococcus | -0.1252359 | 0.11018762 | 0.6359741 |
| Enterobacter | 0.17046353 | 0.16838035 | 0.67945482 |
| Collinsella | -0.2491108 | 0.31775371 | 0.77016586 |
| Lactobacillus | 0.19787031 | 0.26288292 | 0.7864416 |
| Granulicatella | 0.08802643 | 0.12492728 | 0.81254025 |
| Clostridioides | -0.1350552 | 0.19106229 | 0.81254025 |
| Tyzzerella_4 | 0.12037847 | 0.1776203 | 0.83063726 |
| Subdoligranulum | -0.0804733 | 0.13058693 | 0.84679244 |
| Atopobium | -0.0680202 | 0.1134445 | 0.84889231 |
| Flavonifractor | 0.092187 | 0.16492173 | 0.86160346 |
| Bifidobacterium | -0.1227512 | 0.22505311 | 0.86903937 |
| Achromobacter | 0.04753048 | 0.08919447 | 0.87545406 |
| Enterococcus | 0.13413724 | 0.26281534 | 0.88246111 |
| Bacteroides | 0.15077322 | 0.32832103 | 0.88846321 |
| Erysipelatoclostridium | 0.09120415 | 0.22063724 | 0.90768751 |
| Streptococcus | 0.04890436 | 0.15518274 | 0.93527558 |
| Eggerthella | -0.0795947 | 0.26836073 | 0.93841018 |
| Variovorax | -0.0224958 | 0.10173828 | 0.95752894 |
| Finegoldia | -0.0149318 | 0.08684659 | 0.96652483 |
| Peptoniphilus | -0.0183694 | 0.14277744 | 0.97368014 |
| Blautia | 0.03879694 | 0.25776656 | 0.97368014 |
| Ruminiclostridium_5 | -0.0146591 | 0.11865581 | 0.97368014 |
| Veillonella | -0.0262225 | 0.27577581 | 0.97368014 |
| Corynebacterium_1 | 0.00684779 | 0.11610203 | 0.97808301 |
| Rothia | 0.01083184 | 0.1873717 | 0.97808301 |
| Actinomyces | 0.00254629 | 0.23436969 | 0.99798934 |
| Negativicoccus | -0.0003004 | 0.11913268 | 0.99798934 |

*The participants without the given indication were used as the reference group*

D. Eczema with sensitization at 5 years

| **feature** | **coef** | **stderr** | **q (adjusted p)** |
| --- | --- | --- | --- |
| Haemophilus | 1.12448597 | 0.27338193 | 0.00115678 |
| Actinomyces | 0.57849078 | 0.30642745 | 0.32762847 |
| Anaerococcus | -0.2630284 | 0.14306443 | 0.35249866 |
| Subdoligranulum | 0.29828608 | 0.17083261 | 0.37641553 |
| Staphylococcus | 0.40597663 | 0.23969185 | 0.40052375 |
| Finegoldia | -0.1898898 | 0.11306778 | 0.40052375 |
| Atopobium | -0.2359241 | 0.14560595 | 0.41711694 |
| Faecalibacterium | 0.21511083 | 0.13276878 | 0.41711694 |
| Negativicoccus | -0.2470546 | 0.15323361 | 0.4188606 |
| Parabacteroides | -0.3946904 | 0.26612354 | 0.48929261 |
| Flavonifractor | -0.3008857 | 0.20902132 | 0.49738969 |
| Burkholderia.Caballeronia.Paraburkholderia | -0.1948795 | 0.13851836 | 0.51131615 |
| Escherichia.Shigella | -0.6366558 | 0.45834943 | 0.52022195 |
| Veillonella | 0.47823269 | 0.36111465 | 0.5466031 |
| Achromobacter | -0.1387798 | 0.11713163 | 0.62972128 |
| Rothia | 0.26596309 | 0.24575948 | 0.65979496 |
| Tyzzerella_4 | 0.25295575 | 0.23146667 | 0.65979496 |
| Clostridioides | -0.2611574 | 0.25128117 | 0.66848936 |
| Curvibacter | 0.15285237 | 0.14701138 | 0.66848936 |
| Bifidobacterium | -0.303477 | 0.29846714 | 0.67825168 |
| Granulicatella | 0.16152578 | 0.16432552 | 0.68118777 |
| Sphingomonas | -0.2239802 | 0.23103375 | 0.68331407 |
| Enterobacter | -0.214613 | 0.22011099 | 0.68331407 |
| Variovorax | -0.1193536 | 0.13412261 | 0.72154102 |
| Gemella | 0.11301593 | 0.16283079 | 0.79708846 |
| Blautia | 0.23572507 | 0.34299399 | 0.79755196 |
| Peptoniphilus | -0.1236425 | 0.18447075 | 0.79802873 |
| Corynebacterium_1 | 0.10087865 | 0.15265125 | 0.79910576 |
| Pseudobutyrivibrio | 0.0920897 | 0.13997625 | 0.79910576 |
| Streptococcus | 0.12620348 | 0.20333775 | 0.82734873 |
| Klebsiella | -0.201155 | 0.39251309 | 0.86794779 |
| Ruminiclostridium_5 | -0.0781141 | 0.15612141 | 0.86990329 |
| Collinsella | -0.1745452 | 0.41733046 | 0.88035246 |
| Lactobacillus | 0.11220081 | 0.34249821 | 0.93794885 |
| Eggerthella | -0.0698476 | 0.35145624 | 0.95590961 |
| Pseudomonas | -0.0212468 | 0.10829492 | 0.95590961 |
| Clostridium_sensu_stricto_1 | -0.0572519 | 0.34227914 | 0.95972698 |
| Akkermansia | -0.0349584 | 0.20171968 | 0.95972698 |
| Erysipelatoclostridium | 0.03052749 | 0.29024875 | 0.97534117 |
| Bacteroides | 0.01599888 | 0.43068089 | 0.99438235 |
| Enterococcus | 0.01082698 | 0.34294591 | 0.99438235 |
| Halomonas | -0.0006015 | 0.11356231 | 0.9977678 |

*The participants without the given indication were used as the reference group*

E. Asthma at 5 years

| **feature** | **coef** | **stderr** | **q (adjusted p)** |
| --- | --- | --- | --- |
| Ruminiclostridium_5 | 0.41792155 | 0.17478325 | 0.15379567 |
| Actinomyces | -0.6795398 | 0.34524401 | 0.29667819 |
| Parabacteroides | 0.53068407 | 0.29952854 | 0.38648485 |
| Sphingomonas | 0.4248416 | 0.26144931 | 0.45769241 |
| Streptococcus | 0.34964781 | 0.22797411 | 0.48171071 |
| Corynebacterium_1 | 0.22126908 | 0.17216769 | 0.58771966 |
| Tyzzerella_4 | -0.2995845 | 0.26136616 | 0.65546144 |
| Flavonifractor | 0.24208932 | 0.23749697 | 0.70222169 |
| Halomonas | -0.1286587 | 0.12794635 | 0.70222169 |
| Lactobacillus | -0.3591203 | 0.38891352 | 0.72911741 |
| Blautia | -0.3484816 | 0.38299466 | 0.7324251 |
| Atopobium | -0.1395883 | 0.16499027 | 0.75392818 |
| Achromobacter | -0.1092322 | 0.13221904 | 0.75691738 |
| Enterococcus | -0.3137287 | 0.38582587 | 0.76360902 |
| Granulicatella | -0.1460523 | 0.18591806 | 0.77530856 |
| Erysipelatoclostridium | -0.2576508 | 0.32764559 | 0.77530856 |
| Collinsella | 0.34294827 | 0.47082921 | 0.79111209 |
| Pseudomonas | -0.0896329 | 0.12252907 | 0.79111209 |
| Bacteroides | 0.3511582 | 0.48670641 | 0.79152061 |
| Pseudobutyrivibrio | -0.1085311 | 0.15781783 | 0.81645309 |
| Burkholderia.Caballeronia.Paraburkholderia | 0.10755042 | 0.15892675 | 0.81831574 |
| Enterobacter | 0.14771222 | 0.24828842 | 0.87031612 |
| Subdoligranulum | -0.1023087 | 0.1939528 | 0.90396741 |
| Eggerthella | -0.1949451 | 0.39605925 | 0.92021884 |
| Veillonella | -0.1214577 | 0.40913769 | 0.97291271 |
| Variovorax | -0.0553104 | 0.15116389 | 0.97291271 |
| Haemophilus | -0.0923843 | 0.31470605 | 0.97291271 |
| Akkermansia | 0.07103366 | 0.23336767 | 0.97291271 |
| Gemella | -0.0418626 | 0.18431372 | 0.97890126 |
| Staphylococcus | 0.04612669 | 0.27143387 | 0.97890126 |
| Anaerococcus | -0.0395364 | 0.1632415 | 0.97890126 |
| Finegoldia | 0.02115612 | 0.12835945 | 0.97890126 |
| Peptoniphilus | 0.0379407 | 0.20896128 | 0.97890126 |
| Clostridioides | -0.0631938 | 0.28421117 | 0.97890126 |
| Escherichia.Shigella | -0.0736213 | 0.51863473 | 0.98100503 |
| Bifidobacterium | 0.03928261 | 0.33562 | 0.9838423 |
| Clostridium_sensu_stricto_1 | -0.0450466 | 0.38195189 | 0.9838423 |
| Negativicoccus | -0.0194562 | 0.17386956 | 0.9838423 |
| Rothia | 0.02281344 | 0.27780237 | 0.9845397 |
| Faecalibacterium | -0.0061372 | 0.1506277 | 0.9845397 |
| Curvibacter | 0.01251746 | 0.16767882 | 0.9845397 |
| Klebsiella | 0.00161639 | 0.44287611 | 0.99955802 |

*The participants without the given indication were used as the reference group*

F. Asthma with sensitization at 5 years

| **feature** | **coef** | **stderr** | **q (adjusted p)** |
| --- | --- | --- | --- |
| Faecalibacterium | 0.4310758 | 0.1863145 | 0.17222566 |
| Curvibacter | 0.40696077 | 0.20627377 | 0.29278387 |
| Enterococcus | -0.8233904 | 0.48110788 | 0.40442305 |
| Ruminiclostridium_5 | 0.31692758 | 0.21935361 | 0.53073332 |
| Collinsella | 0.79875317 | 0.5864866 | 0.57679119 |
| Actinomyces | -0.5251994 | 0.43291991 | 0.65813679 |
| Atopobium | -0.2483153 | 0.20544466 | 0.65840119 |
| Bacteroides | -0.6634051 | 0.60565775 | 0.6895302 |
| Anaerococcus | -0.2097902 | 0.20218148 | 0.71892906 |
| Pseudobutyrivibrio | 0.19397782 | 0.19702961 | 0.72440864 |
| Corynebacterium_1 | 0.19622485 | 0.21491309 | 0.76222185 |
| Streptococcus | 0.25454568 | 0.28626963 | 0.76222185 |
| Tyzzerella_4 | -0.2496591 | 0.32633725 | 0.80202309 |
| Flavonifractor | 0.22124231 | 0.29506584 | 0.80976666 |
| Veillonella | -0.3535526 | 0.50959923 | 0.83088242 |
| Pseudomonas | 0.10369346 | 0.15245921 | 0.83497386 |
| Peptoniphilus | -0.1490269 | 0.25990036 | 0.89884542 |
| Eggerthella | -0.2367846 | 0.49494927 | 0.91417984 |
| Gemella | 0.1051065 | 0.22946091 | 0.91417984 |
| Subdoligranulum | 0.11048292 | 0.24160741 | 0.91417984 |
| Negativicoccus | -0.1002726 | 0.21657924 | 0.91417984 |
| Burkholderia.Caballeronia.Paraburkholderia | -0.0868732 | 0.19561525 | 0.91417984 |
| Halomonas | 0.06989583 | 0.15992731 | 0.91417984 |
| Sphingomonas | 0.13674241 | 0.32579821 | 0.91422756 |
| Haemophilus | 0.16628937 | 0.39409773 | 0.91422756 |
| Klebsiella | 0.22578568 | 0.55299155 | 0.91634779 |
| Lactobacillus | -0.1446393 | 0.48247471 | 0.94399093 |
| Blautia | -0.1458371 | 0.4834213 | 0.94399093 |
| Enterobacter | -0.0934478 | 0.31043742 | 0.94399093 |
| Bifidobacterium | -0.1131008 | 0.42100869 | 0.95341869 |
| Rothia | 0.08621449 | 0.34673297 | 0.95748794 |
| Granulicatella | 0.05455618 | 0.23177599 | 0.95748794 |
| Akkermansia | -0.0631579 | 0.28414626 | 0.96077203 |
| Parabacteroides | 0.06254832 | 0.3760252 | 0.9718253 |
| Finegoldia | 0.02465261 | 0.15990171 | 0.9764069 |
| Staphylococcus | 0.03344206 | 0.33900453 | 0.98393659 |
| Erysipelatoclostridium | -0.0484319 | 0.4088595 | 0.98393659 |
| Achromobacter | 0.01299153 | 0.16532378 | 0.98393659 |
| Escherichia.Shigella | 0.03557509 | 0.6474119 | 0.98792057 |
| Variovorax | -0.0085104 | 0.18914354 | 0.9927248 |
| Clostridioides | -0.0073073 | 0.35450862 | 0.99586087 |
| Clostridium_sensu_stricto_1 | 0.00311682 | 0.48217326 | 0.99730855 |

*The participants without the given indication were used as the reference group*
